# Immunohistochemical subtype and its relationship with 5-year overall survival in breast cancer patients

**DOI:** 10.3332/ecancer.2023.1509

**Published:** 2023-02-16

**Authors:** Pablo Aldaz-Roldán, Diego F Pardo-Vásquez, Geanella N Chamba-Morales, Daniel F Aguirre-Reyes, Johana M Castillo-Calvas, Gabriela Noblecilla-Arévalo

**Affiliations:** 1 Department of Health Sciences, Universidad Técnica Particular de Loja, Loja 110107, Ecuador; 2 Department of Radiotherapy and Nuclear Medicine, Hospital de la Sociedad de Lucha Contra el Cáncer SOLCA Núcleo de Loja, Loja 1101256, Ecuador; 3 Department of Tumor Registry, Hospital de la Sociedad de Lucha Contra el Cáncer SOLCA Núcleo de Loja, Loja 110105, Ecuador

**Keywords:** breast cancer, survival, immunohistochemical subtype

## Abstract

**Background:**

Breast cancer (BC) is the malignant tumour that has been most frequently diagnosed, being the second most common cancer worldwide and the most frequent in women.

**Objective:**

To analyse the probability of 5-year overall survival according to age, stage of disease, immunohistochemical subtype, histological grade and histological type in patients with BC.

**Methodology:**

Operational research that used a cohort design of patients diagnosed with BC at the SOLCA Núcleo de Loja-Ecuador Hospital from 2009 to 2015 and with follow-up until December 2019. Survival was estimated with the actuarial method and Kaplan–Meier method, and, for multivariate analysis, the proportional hazards model or Cox regression was used to estimate the adjusted Hazard Ratios (HRs).

**Results:**

Two hundred and sixty-eight patients were studied. Mean overall survival was 4.35 years (95% confidence interval (95% CI): 40.20–4.51) and 66% survived to 5 years. The main predictors of survival were advanced stage of disease (III–IV) (HR = 7.03; 95% CI: 3.81–12.9); patients human epidermal growth factor receptor 2-neu (HER2-neu) overexpressed (HR = 2.26; 95% CI = 1.31–4.75) and triple negative (HR = 2.57; 95% CI = 1.39–4.75). The other variables were not significant.

**Conclusions:**

The results show a higher mortality associated with higher clinical stage, more aggressive histological grades and immunohistochemical subtype HER2-neu overexpressed and triple negative tumours.

## Core purpose

First, the objective is to perform survival analysis that provides knowledge of the factors that are associated with a greater survival in patients with breast cancer (BC) treated in the Oncology Unit of the SOLCA Núcleo de Loja, since this region, according to The National Tumour Registry, is one of the provinces in Ecuador with higher incidence (25.9). The results imply that early stages of the disease and molecular subtype luminal are factors that positively influence survival.

## Introduction

According to GLOBOCAN, the highest incidence of BC occurs in Australia and New Zealand (95.5), North, West and South Europe (90.6–90.7) and North America (89.4). In South America, the incidence has been determined at 56.4. However, the data are variable, presenting incidences as high as 73.1 in Argentina, 65.1 Uruguay, 58.5 in Paraguay and 52.6 in Venezuela. In Ecuador, it has been calculated that the incidence is 38.2, becoming the leading cause of cancer in the country [1].

One of the factors that most influence survival is the stage of the disease at diagnosis. The stage and survival have an inversely proportional relationship. According to statistics from the National Cancer Institute’s database, the 5-year survival for stage I is 100%, and 19% for stage IV [2] and these results have been corroborated in other publications [3].

Currently, the molecular subtypes of BC (luminal A and B, triple negative and human epidermal growth factor receptor 2-overexpressed (HER2-overexpressed) are also included as elements that strongly influence survival, since they completely modify the treatment schemes [4]. Most publications have determined that luminal-type expression patterns have better survival because they are more similar to normal breast tissue; on the other hand, triple negative patterns (without expression of oestrogen receptors (ER), progesterone receptors (PR) and HER2 receptors) and HER2-overexpressed have a worse prognosis [5, 6].

In Latin America, there are few published studies that have assessed the influence of prognostic factors, such as age, clinical stage, molecular subtype, histological grade or histological type, on overall 5-year survival in patients with BC [7]. In this sense, the present research is one of the first multivariate analyses of global survival at 5 years in patients with BC in South America, which determines the risk of death considering the variables mentioned above.

## Materials and methods

### Design

This is a non-parametrical and operative investigation that used a cohort design with all patients diagnosed with BC at the SOLCA Núcleo hospital in Loja-Ecuador from 2009 to 2015 with follow-up until December 2020.

### Context

The study was developed at the Hospital SOLCA Núcleo de Loja which is the referral unit for patients for oncological diagnosis, treatment and follow-up in southern Ecuador. This centre has an area in which the data of all patients with a histological diagnosis of cancer are collected, known as the ‘SOLCA Núcleo de Loja Tumor Registry’, covering the province located in southern Ecuador, with approximately 2.56% of the Ecuadorian population. In this registry, staff are continuously trained in the collection of patient data in accordance with the recommendations of the International Agency for Research on Cancer.

### Population and sample

The type of sampling was non-probabilistic and only patients who fulfilled the inclusion criteria were considered: complete data in their clinical records, patients with BC with clinical stage 0–IV, complete treatment, existence of clinical record with histopathological and immunohistochemical or fluorescent in situ hybridisation (FISH) report. Male patients were excluded.

We obtained the data of 348 cases of women with BC registered between 01 January 2009 and 31 December 2015. Of these, 323 (92.81%) were histologically conﬁrmed cases, of these, 55 were excluded from the study for incomplete data. Finally we work with 268 patients with BC.

### Data source

The information was obtained from SOLCA Núcleo de Loja Tumor Registry`s database, which is a population-based registry that has been collecting cases programmatically from public and private institutions of all patients diagnosed with cancer in the province of Loja-Ecuador since 1997. This registry collects and refines the information, so that the database contains information from both public and private institutions of all biopsy samples or surgical pieces with histopathological confirmation of cancer inside and outside the province of patients residing in Loja, identifying the patient, determining the date of diagnosis. This database is updated until 2015 with sociodemographic and clinical information of the patients. Access to information is given with prior permission from the Executive Presidency of SOLCA Loja whenever it is used for research or publication purposes.

### Variables

The variables considered for the analysis were: age in years (in age groups <40; 40–49; 50–59; 60–69; 70–79; and >80), stage 0–IV determined according to Tumour size, Nodes and Metastasis of the American Joint Committee on Cancer version 8. Immunohistochemical subtype according to ER, PR and HER2 status: luminal regarded as ER+ and/or PR+ and HER2−; HER2-overexpressed (HER2+++) and triple negative (ER−, PR− and HER2−). Hormonal receptor status and HER2 overexpression were recorded from pathology and clinical reports. ER, PR and HER2 status were assessed by means of immunohistochemistry (IHC). The cutoff for ER and PR positivity used was >10%. Tumours were considered as HER2+ when cells presented strong membrane staining (3+). Tumours exhibiting 0 or 1+ staining for HER2 protein overexpression were considered to be HER2−. In cases of equivocal membrane staining (score 2+) for HER2, (FISH was used to evaluate gene ampliﬁcation.

Histological grade was described according to Bloom–Richardson score in: Grade 1–3; histological type was considered as ductal, lobular and other.

To establish 5-year survival, the initial date was defined as the diagnosis established according to the first histopathological report of the patient, obtained by biopsy. The end date was defined as the date of death, considered as the event to be studied for the survival analysis, which was established according to the tumour registry, and a check was made of all patients according to the death certificate provided by the Civil Registry of Ecuador. In the case of living patients, the cohort date for follow-up was the last control on 31 December 2020, also confirming their living status on the website of the Civil Registry of Ecuador.

The date of last contact was established by searching the medical record and, if this was not available, the patient or her relatives were contacted, and, as a last resort, by searching the civil registry.

### Statistical analysis

To describe the population, frequencies and percentages were used for the qualitative variables and the median and interquartile range (IQR) for the quantitative variables.

The calculation of survival in each of the 5 years was established using the actuarial method for each study variable. To compare the survival time between groups, means and Kaplan–Meier survival curves were estimated, considering the variables: age, stage, molecular subtype, histological grade and histological type. To establish statistical differences in survival according to variables, the Log Rank statistic was used and a two-sided *p*-value < 0.05 was considered significant.

For multivariate analysis, the proportional hazards model or Cox regression was used. The Hazard Ratio (HR) with its respective confidence intervals was established. For data analysis, the SPSS version 24 package was used.

## Results

**Figure 1.**
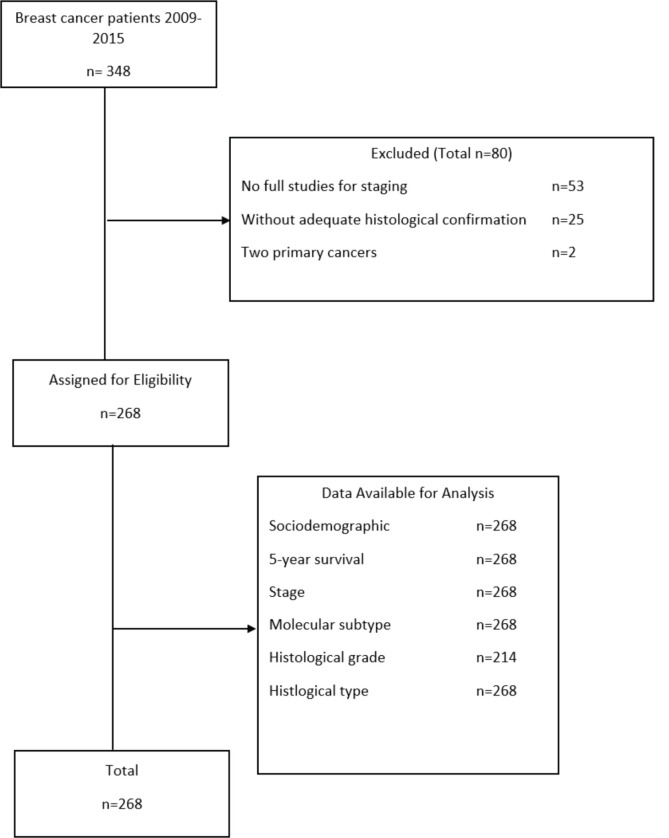
Patient selection flowchart for the study. Made by: Aldaz et al [13].

From 348 patients diagnosed with BC of any denomination, 80 patients were excluded due to non-compliance with the eligibility criteria (Figure 1). The final sample was 268 patients and none were lost during the study.

The mean age of the patients studied was 54.62 years (IQR: 45–64), with a predominance in ages from 40–49 years (31.6%). From the clinical characteristics of the patients upon admission, it was highlighted that 40.3% were stage II, 44.0% had a molecular subtype luminal, 41.0% had histological grade 2 and 97% were ductal type (Table 1).

The actuarial analysis for survival by years showed that, in the first year, the survival was 96% and reached 66% in the 5th year. According to the results obtained in the population studied, survival was higher in patients under 40 years old, with stage 0 and I, with molecular subtype luminal, grade 1 and histological subtype others (mucinous and medullar) (Table 2).

According to the bivariate analysis, a lower survival was found in patients with advanced stage III–IV (mean survival = 3.86; 95% confidence interval (95% CI) = 3.59–4.13) and HER 2 overexpressed (mean survival = 4.11; 95% CI = 3.28–4.40). The rest of the variables did not show statistically significant results (Table 3).

In the Cox regression model, the worse survival predictors presented were: advanced stage (III–IV) HR = 7.03 (95% CI = 3.81–12.9 *p* < 0.001); immunohistochemical subtype HER2 overexpressed HR = 2.26 (95% CI = 1.32–4.75; *p* < 0.001); and molecular sub type triple negative HR = 2.57 (95% CI = 1.39–4.75; *p* < 0.001). The other variables did not show significant differences (Table 4).

**Figure 2.**
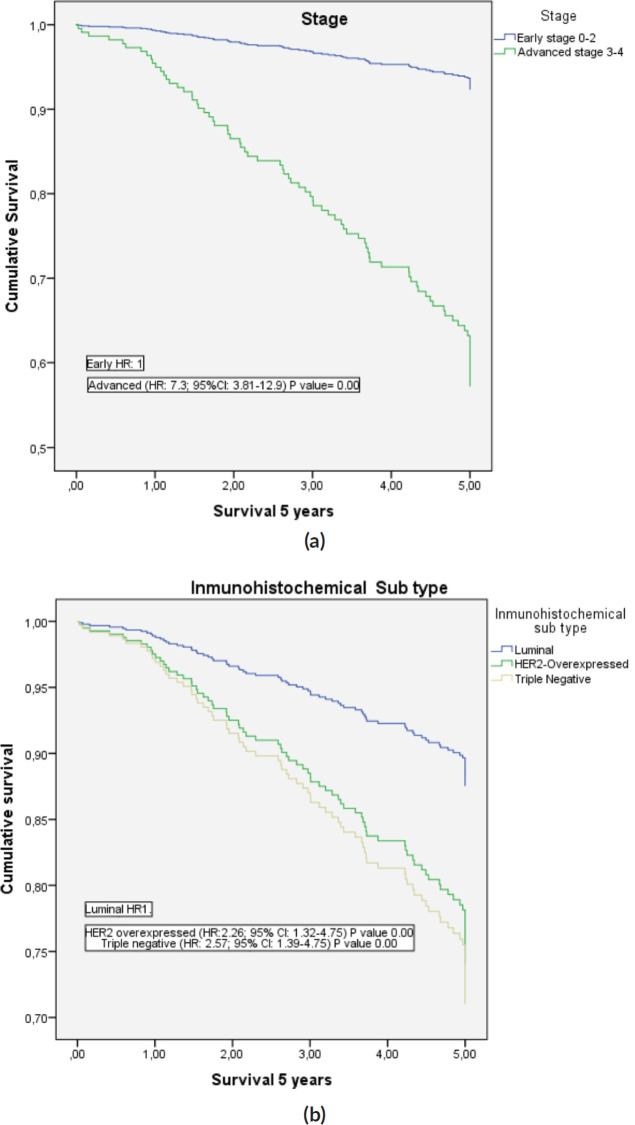
Adjusted 5-year survival of patients with BC. (a): 5-year survival curves as a function of early or advanced stage; (b): Depending on immunohistochemical subtype.

The 5-year survival adjusted for all the factors evaluated was higher in patients under 40 years old, early stage (0–II), luminal sub type, Grade 1 and Ductal (Figure 2 (a and b))

## Discussion

This is the first study in Ecuador that uses the database from the SOLCA Núcleo de Loja Tumour Registry for the analysis of the cohort of patients with BC. It was concluded that the variables that negatively influence overall survival are ages between 70 and 79 years, advanced disease, immunohistochemical subtype HER2-neu and triple negative, moderate and grade 3 of differentiation scale and lobular histology.

One of the relevant characteristics of the studied population is the age of presentation at the time of diagnosis of BC being 40–49 years, within the expected parameters. Therefore, the most important risk factor for the development of cancer is age [8]. Various studies support this result, with age ranges between 41 and 50 years and an average of 53.7 years [9, 13]. The influence of the age of presentation on global survival in this study determined that patients between 70 and 79 years had a higher risk of death compared to the other age groups (HR: 2.69; 95% CI: 0.96–7.52; *p* = 0.06), although the results were not statistically significant. A study with 172,179 BC published similar results presenting a HR of 3.52, in patients older than 79 years [10]. However, the rest of the results are contradictory to those reported in the international literature. Data from the study published by Eric *et al* [11] determined that patients younger than 40 years presented worse prognostic factors when compared with patients older than 60 years, among whom were reported grade 3 (29% versus 17%), oestrogenic receptor negativity (45% versus 23%), multicentric BC (23% versus 5%), triple-negative (32% versus 10%) and a higher proliferation index Ki-67 (25% versus 10%), *p* value < 0.05. Other studies confirm this trend with a greater number of patients [12].

The clinical stage and survival results have an inversely proportional relationship. According to statistics from National Cancer Institute’s database, the 5-year survival for stage I is 100%; for stage II, 85%; for stage III, 58%, and 19% for stage IV [2]. In this study, we present similar results to those mentioned above, with a 5-year survival of 100% for stage 0 and I, 84% for stage II, 60% for stage III and 11% for stage IV.

When evaluating the risk of death, it was determined that patients with advanced stages (III–IV) have a HR of 7.03 (95% CI: 3.81–12.9; *p* = <0.001) compared with patients with early stage (0–II). In the study published by Ferguson *et al* [3], it was indicated that for cumulative survival, in months, only clinical stage, lymph node involvement, metastasis stage and age were significant predictors of death. ‘The patients with T2 tumours, N2, N3 tumours and M1 tumours were 1.72, 2.79, 2.63, and 4.0 times more likely to die than patients with T1, N0/N1 tumours, and M0 tumours, respectively (95% CI: 1.093–2.729 for T2; 1.48–5.25 for N2, 1.17–5.92 for N3; and 1.66–9.64 for M1)’ [3].

The Immunohistochemical subtype that appeared in most of the patients was luminal, with 44%, followed by overexpressed HER2-neu (35.8%) and, to a lesser extent, triple negative (20.1%). Results published by Puig-Vives *et al* [5] determined that the luminal pattern is of predominant appearance, with 68%, followed by HER2-neu overexpressed, with 19.5%, and finally triple negative in 11.8% in a Spanish population. The same trend was published in the study Surveillance, Epidemiology, and End Results that showed a higher proportion of patients with type luminal A 72%. The other subtypes were presented as follows: Luminal B 10.9%, HER2-neu 4.8% and triple negative 11.8% [3]. Other studies showed similar results [9, 14].

The influence of the immunohistochemical subtype on the survival of our patients showed that patients with luminal type had a better prognosis, while patients with HER2-neu overexpressed had a HR of 2.26 (95% CI: 1.32–4.75) and triple negative patients of 2.57 (95% CI: 1.39–4.75). Similar results were published in a study of Spanish patients in which the relative excess risk of death was 1.72 (95% CI: 1.15–2.57) for patients with HER2 overexpressed and 3.16 (95% CI: 2.26–4.41) for triple-negative patients [5]. In the same way, another study of 1,945 patients determined that luminal A tumours had a better prognostic than luminal B (HR: 1.90, 95% CI: 1.33–2.71), HER2-type (HR: 1.36, 95% CI: 0.87–2.12) and basal-like (HR: 1.58, 95% CI: 1.05–2.39). Similar tendencies were observed for both overall and recurrence-free survival [15]. Additionally, this study evidenced that the HER2-neu overexpressed determines a decrease in survival. According to data reported by Slamon *et al* [17], the amplification of the gene HER2/neu is a significant predictor of overall survival and relapse time in patients with BC. This is demonstrated by revealing the cases in which the HER2/neu overexpression is related to a survival shorter than 5 years, compared to those who do not overexpress it. Specifically, they showed a 5-year overall survival in HER2 positive patients of 64% compared to 81% in HER2 negative [16]. These results were corroborated by Cadoo *et al* [18], who described that the positivity of HER2, in relation to overall survival, is a poor prognostic factor independently of other prognostic characteristics such as age, nodal status, tumour size, tumour grade, hormonal receptor status and adjuvant treatment. and finally these findings were also similar to those reported in a recent multicentre study in Latin America in which it was determined that patients with luminal A IHC appeared to have significantly better prognoses of cancer-specific survival and progression-free survival compared with the other subtypes [19]. Making a comparative analysis of the results, the cohort from SOLCA HER2 patients determined that this has a worse prognosis than other Latin cohorts shown in the multicentric study publication by Llera *et al* [19], one possible explication of this could be due to that the cohort from SOLCA HER 2 included patients with clinical stages I to IV, and the last group involved 39 patients with metastatic disease, which can be the reason for that behaviour in the survival and give this results with poor prognosis in the general population, if it is compared with the patients in the Multicentric cohort from the study (*frontiersin*) that not included, patients in stage I and IV, as a result of this, the prognosis of this cohort is better and is not comparable with our cohort, due to if you compare stages with locoregional disease versus metastatic disease you obtain worse prognosis for metastatic disease, independently of HER 2 status.

To a lesser degree of histological differentiation, the behaviour is more aggressive in BC. The data show that the HR is higher in grades 2 and 3 compared to grade 1, and that the probability of survival is higher in tumours of grade 1 compared to grades 2 and 3, similar to the data published by Tot *et al* [20] (grade 2 HR: 2.15; 95% CI: 0.82–5.70 and grade 3 HR: 2.90; 95%CI: 1.05–8.05). This may be due to the fact that patients with grade 2 and 3 usually present tumours with overexpressed or triple negative HER2. This was demonstrated in the study carried out in Borneo in which HER2-neu overexpressed patients had grade 2 in 44.7% of cases and grade 3 in 47.8% of cases, while triple negative patients had grade 2 in 46% of cases and grade 3 in 49% of cases [21]. Other studies showed similar results [5].

Finally, in relation to the survival linked to the histological type, the present study determined that patients with ductal carcinomas had a better prognosis than lobular ones, although the results were not statistically significant (HR: 4.57; 95% CI: 0.57–36.04). These results show a course contrary to those published by the literature in retrospective studies that indicate that the lobular type has a mortality risk lower than ductal [22, 23], as was determined by Cristofanilli *et al* in their study made in 2005 of lobular carcinoma patients.

This study has some limitations, mainly that it was a retrospective study and based on secondary sources. Patients without imaging studies had to be excluded to calculate the stage of the disease. This situation occurred at the beginning of the evaluated period due to the lack of subsidy from the state and the expenses of the studies and treatment were borne by the patients. Nevertheless, its inclusion could have incurred information bias in the evaluation of survival, and, for this reason, our results are as robust as those reported in previous studies. Additionally, factors that have been reported to be associated with 5-year survival, such as surgery and chemotherapy treatment regimen, were not studied because the objective of the present study was to determine the influence of the molecular subtype on survival. However, the information analysed under the focus of an operational investigation included the main prognostic factors that have been analysed to study BC survival.

Within the advantages, it is one of the first studies in the region to analyse the influence that a wide spectrum of variables has on 5-year survival in BC; previous studies have been limited to bivariate analyses, but, in our study, adequate analytical methods were used to analyse survival. Additionally, we consider that, being the first study in Ecuador and of this operative nature in the region, it may represent a baseline for future multicentre studies in which survival in this type of cancer is to be evaluated. Finally, we follow the STROBE guidelines for the proper reporting of a cohort study.

## Conclusions

The results of the study show a higher mortality associated with higher clinical stage, more aggressive histological grades and immunohistochemical subtype HER2-neu overexpressed and triple negative tumours.

## Conflicts of interest

The author(s) declare(s) that there is no conflict of interest regarding the publication of this paper.

## Financial conflicts of interest

The author(s) declare(s) that there is no financial conflict of interest regarding the publication of this paper.

## Ethics policies/statements

This is an operational research that was based on data from clinical histories or from the SOLCA Nucleo de Loja Tumor Registry. Approval by a Human Research Ethics Committee was not necessary; however, the authors maintain the data assured with coding of patients to ensure their confidentiality. The data collected in this work are for the exclusive use of research purposes.

## Informed consent

It was not necessary due to the nature of an operative investigation.

## Data availability

The crude data used to support the findings of this study may be released upon application to the ‘Registro de Tumores SOLCA Loja’, who can be contacted through the corresponding author, or at the following e-mail: rtumores@solcaloja.med.ec.

## Funding

This research was self-financed by the authors.

## Authors’ contributions

Pablo Aldaz-Roldán: Idea, study design, data collection, statistical analysis, data interpretation, draft article writing, critical review of the article and final approval of the version to be published.

Gabriela Noblecilla-Arévalo: Design, data collection, statistical analysis, data interpretation, draft article writing, critical review of the article and final approval of the version to be published.

Daniel F Aguirre-Reyes: Statistical analysis, data interpretation, critical review of the article and final approval of the version to be published.

Geanella N Chamba-Morales: Idea, study design, data collection, statistical analysis and data interpretation.

Diego F Pardo-Vásquez: Idea, study design, data collection, statistical analysis and data interpretation.

Jhoanna M Castillo-Calvas: Idea, data collection, statistical analysis, data interpretation and critical review of the article.

## Supporting foundations

Supported by: SOLCA Núcleo de Loja Hospital and Universidad Técnica Particular de Loja.

## Figures and Tables

**Table 1.** Characterisation of patients with BC.

Variable		Mean	IQR^a^
Age	Value	54.62	45–64
	Min./Max.	28–83	
		*N*	%
Age			
	<40	30	11.2%
	40–49	85	31.6%
	50–59	61	22.7%
	60–69	53	19.7%
	70–79	29	10.8%
	>80	10	3.7%
Stage	Stage 0	3	1.1%
	Stage I	20	7.5%
	Stage II	108	40.3%
	Stage III	98	36.6%
	Stage IV	39	14.6%
Immunohistochemical subtype	Luminal	118	44.0%
	HER2 overexpressed	96	35.8%
	Triple negative	54	20.1%
Histological grade	Grade 1	14	5.2%
	Grade 2	110	41.0%
	Grade 3	85	31.7%
	Unknown	54	22.0%
Histological type	Ductal	260	97.0%
	Lobular	3	1.1%
	Others	5	1.9%

a IQR, Interquartile range

**Made by:** Aldaz *et al* [13].

**Table 2.** Percentage of survival per year.

Variables		Year 0	Year 1	Year 2	Year 3	Year 4	Year 5
General population	*n*% (SE)	96 (1)	89 (2)	84 (2)	79 (3)	73 (3)	66 (3)
Age	<40	100 (0)	93 (5)	86 (7)	83 (7)	79 (8)	79 (8)
	40–49	96 (2)	88 (3)	85 (4)	76 (5)	74 (5)	62 (6)
	50–59	97 (2)	90 (4)	85 (5)	75 (6)	66 (6)	59 (7)
	60–69	92 (4)	89 (4)	87 (5)	87 (5)	79 (6)	75 (7)
	70–79	100 (0)	89 (6)	79 (8)	75 (8)	71 (9)	58 (11)
	>80	90 (9)	90 (9)	80 (13)	80 (13)	70 (14)	70 (14)
	0	100 (0)	100 (0)	100 (0)	100 (0)	100 (0)	100 (0)
Stage	I	100 (0)	100 (0)	100 (0)	100 (0)	100 (0)	100 (0)
	II	100 (0)	98 (1)	96 (02)	93 (2)	91 (03)	84 (4)
	III	98 (1)	91 (3)	84 (4)	77 (4)	70 (5)	60 (6)
	IV	79 (6)	56 (8)	44 (8)	31 (7)	15 (6)	11 (6)
Immunohistochemical subtype	Luminal	98 (1)	95 (2)	91 (3)	90 (3)	84 (3)	76 (5)
	HER2 overexpressed	95 (3)	85 (4)	79 (4)	70 (5)	64 (5)	56 (6)
	Triple negative	94 (3)	85 (5)	80 (5)	70 (6)	64 (7)	61 (7)
Histological grade	Grade 1	100 (0)	93 (7)	93 (7)	93 (7)	93 (7)	93 (7)
	Grade 2	97 (2)	94 (2)	90 (3)	80 (4)	77 (4)	64 (6)
	Grade 3	94 (3)	83 (4)	78 (4)	72 (5)	69 (5)	64 (6)
	Unknown	97 (2)	90 (4)	81 (5)	81 (5)	67 (6)	67 (6)
Histological type	Ductal	97 (1)	89 (2)	84 (2)	78 (3)	73 (3)	65 (3)
	Lobular	67 (27)	67 (27)	67 (27)	67 (27)	67 (27)	67 (27)
	Others	100 (0)	100 (0)	100 (0)	100 (0)	100 (0)	100 (0)

SE, Standard error

**Made by:** Aldaz *et al* [13].

**Table 3.** Mean survival in years for the study population.

Variable		Deceased	Mean survival	95% CI	*p* value (*)
Overall survival		80	4.35	4.20–4.51	
Age	<40	6	4.52	4.14–4.90	0.60
	40–49	27	4.38	4.05–4.60	
	50–59	22	4.32	4.00–4.63	
	60–69	12	4.42	4.02–4.82	
	70–79	10	4.27	3.78–4.77	
	>80	3	4.20	3.21–4.51	
Stage	Early	13	4.86	4.76–4.96	0.00
	Advanced	67	3.86	3.59–4.13	
Immunohistochemical subtype	Luminal	23	4.64	4.46–4.83	0.003
	HER2 overexpressed	37	4.11	3.28–4.40	
	Triple negative	20	4.15	3.77–4.53	
Histological grade	Grade 1	1	4.76	4.33–5.20	0.26
	Grade 2	32	4.48	2.27–4.69	
	Grade 3	28	4.14	3.83–4.46	
	Unknown	19	4.32	3.97–4.66	
Histological type	Ductal	79	-	-	0.26
	Lobular	1	-	-	
	Others	0	-	-	

CI, Confidence interval

(*) Log rank test

**Made by:** Aldaz *et al* [13].

**Table 4.** Model of the proportional risks associated with the survival of patients with BC.

Variable		HR crude	95% CI	*p* value	HR adjusted	95% CI	*p* value
Age	<40	1			1		
	40–49	1.65	0.68–4.00	0.26	1.68	0.69–4.09	0.25
	50–59	1.93	0.78–4.76	0.15	1.74	0.70–4.33	0.23
	60–69	1.15	0.44–3.09	0.76	1.10	0.40–2.99	0.85
	70–79	1.85	0.67–5.08	0.23	2.69	0.96–7.52	0.06
	>80	1.57	0.39–6.26	0.52	2.76	0.67–11.36	0.16
Stage	Early	1			1		
	Advanced	6.66	3.67–12.06	0.00	7.03	3.81–12.9	0.00
Immunohistochemical subtype	Luminal	1			1		
	HER2 overexpressed	2.33	1.38–3.93	0.00	2.26	1.32–4.75	0.00
	Triple negative	2.18	1.19–3.97	0.01	2.57	1.39–4.75	0.00
Histological grade	Grade 1	1			1		
	Grade 2	4.59	0.67–33.60	0.13	1.80	0.24–13.59	0.56
	Grade 3	5.65	0.76–41.56	0.09	2.30	0.31–17.32	0.41
	Unknown	5.27	0.70–39.34	0.11	2.17	0.28–16.64	0.45
Histological type	Ductal	1			1		
	Lobular	1.29	1.79–9.25	0.80	4.57	0.57–36.04	0.15

Made by: Aldaz et al [13].
